# Nerve allografts - a review

**DOI:** 10.1186/1753-6561-9-S3-A77

**Published:** 2015-05-19

**Authors:** Rodrigo Banegas

**Affiliations:** 1 Peripheral Nerve Institute, Denver Health Medical Center, Denver, Colorado, 80204, USA

## 

In axonotmesis, peripheral nerve regeneration, target reinnervation and recovery of function happen remarkably well with a conservative management in most of the cases. Also, substantial axonal regrowth and nerve regeneration can occur with nerve transection injuries after primary end-to-end neurorrhaphy. Nearly similar outcomes can be obtained using a nerve autograft to repair a nerve defect between severed nerve ends. However, regardless of repair strategy, recovery of function after a more proximal neurotmesis is partial at best and catastrophe is not uncommon.

Cajals’ study in 1928 found that in neurotmesis the axonal sprouts emerge from the basal lamina tube and wander within a disorganized mass of perineurial and Schwann cells to form a neuroma. The neuroma is a foundation of abnormal sprouting caused by the interruption of endoneurial tubes in a traumatic nerve injury. Scadding and Thomas in 1983 and later Leis in 2003 described that the nerve sprouts might also turn back and grow in a backward direction. In addition, the nerve sprouting can extend significant distances along the parent nerve and may even backtrack to proximal branches, before innervating novel targets.

In an animal study Graham et al have pointed that the antegrade, target-directed axonal regeneration can be affected by aberrant and dysfunctional regrowth due to a rich in growth-inhibiting chondroitin sulfate proteoglycan. This may contribute to misdirection of axonal regrowth and a poorer functional result bellow the initial expectation of the surgeon and patient. The idea behind this research finding is to degrade the chondroitin sulfate proteoglycan by application of chondroitinase at the site of nerve repair and in this way decrease aberrant axonal growth. The application of this principle in prepared allograft nerve can help in the target directed regrowth and probably in achieving better functional outcomes.

Krekoski et al. have shown that segments of rat sciatic nerve of 1.5 cm in length were made acellular by repeated freeze thaw cycles and then bathed en bloc in a chondroitinase ABC solution for 16 hours. Then, the en bloc chondroitinase treatment of acellular nerve grafts effectively degraded CSPG (chondroitin sulfate proteoglycans) without compromising the basal lamina scaffold or dislocating its laminin content.

Cho et al showed that this method is effective and favorable when compared with auto graft and nerve tubes in treating nerve gap of 5 to 50 mm in a rat study.

Tang et al in an animal study demonstrated that the use of acellular nerve allograft demonstrated equal functional recovery when compared to reversed autograft and superior recovery compared to the cabled nerve autograft. Even though functional recovery after a nerve injury in humans is affected by many other factors compared with rats, the idea of using acellular allograft nerve is a resource to be considered.

Finally, Neubauer et al in a rodent model found that the histological examination of acellular nerve graft revealed much better structural preservation and axonal growth throughout the ChABC (chondroitinase ABC) grafts. Numerous axons were host distal nerves and many of these regenerated axons were myelinated. In addition, the amount of aberrant retrograde axonal growth (originating near the proximal suture line) was markedly reduced by repair with ChABC grafts.

